# Strengthening community midwives to tackle gender-based vulnerabilities during the 2022 floods in Pakistan—lessons learned for future natural disasters

**DOI:** 10.1186/s44263-023-00002-9

**Published:** 2023-07-31

**Authors:** Iram Kamran, Ali Mohammad Mir

**Affiliations:** Population Council, 3rd Floor, NTC Building (North), F-5/1, Islamabad, Pakistan

**Keywords:** Floods, Pakistan, Community midwives, Maternal health, Vulnerability

## Abstract

Several countries across different continents experienced severe flooding in 2022, including Pakistan. Given the greater frequency and affliction of natural disasters like flooding in an increasing number of countries, we share lessons from the experience of Pakistan in helping the most vulnerable populations through community-based health workers.

## Background

Nearly 33 million people—approximately 16% of the total population of Pakistan—have been adversely impacted by the floods that hit the country at the beginning of June 2022 [1]. The flooding created an unprecedented humanitarian crisis in which 1700 people lost their lives and 1.7 million homes were wiped out. Furthermore, 22,000 schools were damaged, and over 1460 health facilities were either partially or completely destroyed [2]. According to the Population Council’s estimates, the floods have affected 5.1 million children and 6.1 million pregnant women [3]. Those most affected are disadvantaged rural communities who already face extreme poverty, with physical and financial constraints making access to health services an even greater challenge [4].

While natural disasters affect everyone, societal, cultural, economic, and biological factors disproportionately increase the impact on vulnerable groups such as women and girls and the economically disadvantaged [5]. Global evidence shows that compared to men, women are more adversely affected by natural disasters.

Gender inequity limits women’s access to education and resources, as well as their decision-making authority, and natural disasters increase their vulnerabilities. This is due to the added pressure on them to look after the injured and sick, in addition to performing their household duties. Evidence suggests that women living through natural disasters have more miscarriages, premature deliveries, uterine growth retardation, low birth weight infants, and unplanned and unwanted pregnancies [6]. A reason could be that emergency healthcare organized at the time of natural disasters is focused on treating acute injuries and outbreaks. Emergency response efforts often overlook the reproductive needs of women and fail to appoint mid-level community health providers like obstetricians and gynecologists as frontline workers [5].

As natural disasters result in the destruction of healthcare facilities, breakdown of the supply chain of medicines and contraceptives, and diminished financial resources, this leads to limited access to health services and supplies, including for sexual and reproductive health (SRH) [7]. In this scenario, the potential of community-based health workers such as lady health workers (LHWs) and community midwives (CMWs) as frontline workers can and needs to be capitalized upon. LHWs, introduced in 1994, are part of the formal health system and are primarily responsible for providing preventive healthcare to around 200 households in rural areas. CMWs were introduced in Pakistan as a community-based cadre in 2006 with the aim of providing SRH services mainly to women living within their vicinity. After 2 years of training, they are supported by the government to establish clinics in their residential villages, termed birth stations, where they provide maternal care services. Although trained and deployed by the government, CMWs are not government employees and, therefore, not part of the public sector health system.

Having closely collaborated with CMWs, the Population Council, an international nongovernmental, nonprofit research and technical organization working in the population and health field, has observed and supported their critical role in providing disaster response services, most importantly the provision of services focused on women living in affected communities. This Comment draws on our experiences in collaborating with CMWs in rural Pakistan.

## Rediscovering the role of CMWs in disaster relief efforts in Pakistan

Next to LHWs, CMWs are the main source of and point of contact for reproductive healthcare for rural women in Pakistan. Our recent study reported that the majority of CMWs provide their services under difficult conditions such as poorly maintained buildings, equipment, and other infrastructure. They do not receive any public sector medicines or other supplies [8]. Natural disasters exacerbate these conditions in an already fragile health system.

The recent floods damaged the CMWs’ homes and birth stations, service rooms, equipment, and medical supplies. While government agencies, donors, and other organizations were quick to set up temporary medical camps for the treatment of medical emergencies, CMWs did not feature in any of the relief activities since they are not part of the formal health system. As a result, their services to women in need of antenatal and postnatal care and safe child delivery were severely hampered.

To engage CMWs in emergency response activities, the Population Council focused on enabling 106 CMWs to become operational in 4–6 weeks in some of the worst-hit districts in Sindh and Balochistan, the provinces most affected by the floods. The districts were identified in collaboration with the flood emergency response authorities of the provincial governments. A rapid needs assessment carried out by the Population Council provided a list of essential supplies such as medicines, contraceptives, infection prevention, child delivery kits, hygiene products, and equipment including delivery tables, and weighing scales. This was followed by a quick refresher training by the Population Council trainers focused on reproductive health issues that women are confronted with, including infection prevention and prevention from vector and water borne diseases. After 6 weeks, under makeshift conditions, CMWs were able to deliver babies in aseptic conditions and to prevent unwanted pregnancies by providing information on family planning.

They also provided emotional support to women who had experienced trauma due to the destructive floods. In a short span of 2 months, 55 CMWs had catered to 792 family planning clients and conducted 479 deliveries, while 664 women received antenatal and 368 postnatal care.

## Recommendations for building resilient community health systems during natural disasters

Drawing from these experiences with CMWs during the catastrophic floods, we propose that in order to mitigate gender-based vulnerabilities in disaster situations, health-related needs of women and children should be addressed as a priority through strengthening community-based health systems. District-specific risk mitigation and resilience plans should include rebuilding community-based healthcare systems by supporting health workers such as CMWs. In order for them to be effective, routine trainings should include sessions on disaster preparedness and management, as well as mental health. They should receive supportive supervision, regular supplies of medicines, and periodic refurbishment of their facilities and maintenance or replacement of equipment. Apart from providing essential SRH care, CMWs can also play a key role in disaster risk reduction by initiating health education sessions. They can prove to be effective communicators as they are aware of local dynamics and are trusted by their communities. They can also apprise health authorities about specific community needs and provide early information on potential disease outbreaks and other notifiable conditions for more targeted and customized relief efforts. Moreover, there should be enhanced collaboration and task sharing between LHWs and CMWs as both can synergistically help women and children to overcome accessibility and affordability hurdles during disaster situations.

## Conclusions

The floods brought to the fore important lessons learned for future disaster management planning in Pakistan and elsewhere. Community health workers are the backbone of the health system in rural Pakistan and not only serve people within their communities but also link them to the formal health system. By helping women and children, CMWs played a crucial role in reducing gender-specific vulnerabilities during the recent floods in Pakistan. In order to respond to similar situations should they arise in the future, community health systems need strengthening in general and particularly to provide reproductive health services optimally.

## Data Availability

Not applicable.

## Abbreviations

CMWs: Community midwives
LHW: Lady health worker
SRH: Sexual and reproductive health
